# Targets, models and challenges in osteoarthritis research

**DOI:** 10.1242/dmm.016881

**Published:** 2015-01

**Authors:** Sarah Thysen, Frank P. Luyten, Rik J. U. Lories

**Affiliations:** 1 Laboratory of Tissue Homeostasis and Disease, Skeletal Biology and Engineering Research Center, KU Leuven, 3000 Leuven, Belgium.; 2 Skeletal Biology and Engineering Research Center, KU Leuven, 3000 Leuven, Belgium.; 3 Division of Rheumatology, University Hospitals Leuven, KU Leuven, 3000 Leuven, Belgium.

**Keywords:** Osteoarthritis, Cartilage, Bone, Animal models

## Abstract

Osteoarthritis is a chronic degenerative disorder of the joint and represents one of the most common diseases worldwide. Its prevalence and severity are increasing owing to aging of the population, but treatment options remain largely limited to painkillers and anti-inflammatory drugs, which only provide symptomatic relief. In the late stages of the disease, surgical interventions are often necessary to partially restore joint function. Although the focus of osteoarthritis research has been originally on the articular cartilage, novel findings are now pointing to osteoarthritis as a disease of the whole joint, in which failure of different joint components can occur. In this Review, we summarize recent progress in the field, including data from novel ‘omics’ technologies and from a number of preclinical and clinical trials. We describe different *in vitro* and *in vivo* systems that can be used to study molecules, pathways and cells that are involved in osteoarthritis. We illustrate that a comprehensive and multisystem approach is necessary to understand the complexity and heterogeneity of the disease and to better guide the development of novel therapeutic strategies for osteoarthritis.

## Introduction

**Fig. 1.**
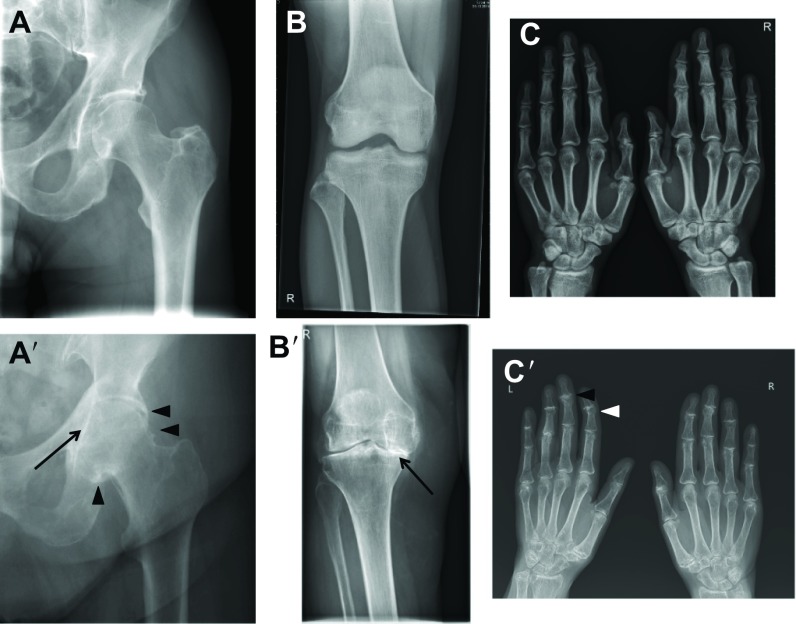
**X-ray radiographic images showing structural alterations of the joints that are most commonly affected by OA.** (A) Normal and (A′) severely affected joint of the hip, characterized by joint space narrowing (arrow) and osteophyte (bony outgrowth) formation (arrowheads). (B) Normal and (B′) severely affected joint of the knee, showing alterations in the medial compartment, with almost complete disappearance of the joint space (arrow) and lateral involvement. (C) Normal and (C′) severely affected joint of the hand. The panel shows the distal interphalangeal joint with disappearance of the joint space and small osteophyte formation (arrowheads).

Osteoarthritis (OA) is a chronic and progressive joint disorder characterized by structural damage to one or more joints that can be recognized on X-ray imaging (Fig. 1). With worldwide estimates that over 10% of the population above 60 years is affected by OA, the impact of this health problem is still underestimated (Hunter et al., 2014). The course of the disease is highly variable between affected individuals: single or multiple joints can be involved and symptoms can vary from mild to severe joint pain and stiffness, often leading to loss of joint function and permanent disability. The prevalence of OA increases with age. Genetic as well as acquired factors, which include joint trauma and life-style-associated risk factors (such as obesity and excessive joint use in occupational or leisurely activities) can contribute to the onset and progression of the disease (Bijlsma et al., 2011; Hunter and Felson, 2006; Lane, 2007). The socioeconomic impact of OA is high and, because aging and obesity are increasing in the population, costs for OA management will also likely increase in the future (Lawrence et al., 2008; Hunter et al., 2014). Current treatment options for OA are limited. In addition to physiotherapy, regular exercise and weight loss, pharmacological interventions are restricted to symptomatic relief with local (intra-articular) injections of corticosteroids and/or systemic administration of analgesics and non-steroidal anti-inflammatory drugs (NSAIDs) (Berenbaum, 2008; Bijlsma et al., 2011). In the most severe cases, osteotomy (a surgical intervention aimed at changing the load pattern in the joint by altering leg alignment) or joint prosthesis (replacing the joint with an artificial device to take over its function and reduce pain) seem to be the only options to partially restore joint functionality and improve quality of life.

Joints are complex organs in which different tissues functionally cooperate to allow movement between the bones of the skeleton, while at the same time limiting the degree and the axes of movement. In the joint, a thin layer of articular cartilage caps the hard and calcified bones. This specialized tissue is rich in extracellular matrix components that attract abundant water molecules, which absorb and translate to the bones the compressive loading forces that are applied to the joint during movements. The joint cavity is further lined by the synovium, a thin connective tissue that produces the lubricating synovial fluid. Finally, ligaments and capsules provide further strength to the joint. A classical view considers OA primarily as a disease of the articular cartilage. Fibrillations and ulcerations, loss of extracellular matrix and cell death occur in this tissue. Damage in the articular cartilage is typically characterized by fissures in its superficial layer that gradually extend into the deeper layers and finally lead to severe loss of cartilage structure and volume. This will then result in secondary changes to the underlying (subchondral) bone and to other tissues of the joint. However, recent observations suggest that OA should be approached as a failure of the entire joint organ and that early disease-related changes can be detected both in the cartilage and in the subchondral bone (Brandt et al., 2008; Brandt et al., 2006; Lories and Luyten, 2011). OA can result from the dysregulation of a complex set of biomechanical and biochemical interactions between multiple structures, which can disrupt the normal homeostasis of the joint (Loeser et al., 2012; Lories, 2011). For example, progressive cartilage loss, subchondral bone remodelling, formation of osteophytes (bony outgrowths) at the joint margins and synovial inflammation (synovitis) are among the processes that characterize OA pathophysiology and which will eventually contribute to increasing joint pain and functional impairment. Although inflammation can take part in the osteoarthritic processes, it is not the dominant driving force of this disease. Other joint diseases, such as rheumatoid arthritis, are driven by inflammation in the first place, whereas damage to cartilage and bone is a secondary phenomenon. The tissues of the joint work together in order to maintain joint homeostasis. Failure in even a single component of the joint can compromise its functionality and progressively lead to the failure of the whole organ (Fig. 1) (Lories and Luyten, 2011).

Further understanding of the molecular and cellular basis of OA is fundamental to guide the identification of new therapeutic targets and the development of specific strategies and interventions. Therefore, a whole range of *in vitro* and *in vivo* systems are currently used to study different aspects of joint physiology in health and disease. Here, we review some of the targets that have emerged from the study of joint function and from genetic association studies. We also discuss recent progress in OA research, with special attention to discoveries made by transcriptomic, proteomic and epigenomic approaches. This provides a platform to discuss how current technologies might help the development of new therapeutic approaches in OA and to identify some of the challenges related to the translation of basic OA research into the clinic.

## Searching for targets and therapies in OA

**Fig. 2.**
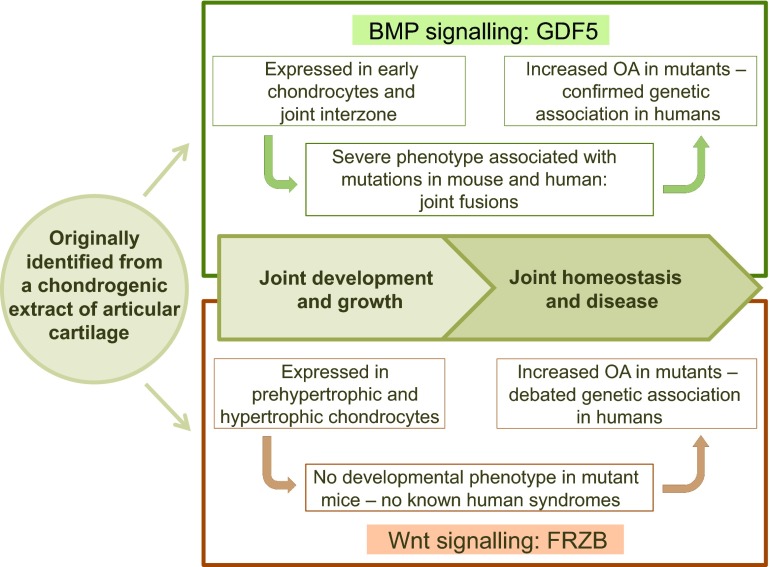
**Different steps in the discovery of two factors that play a key role in joint physiology and pathophysiology.** Growth and differentiation factor-5 (GDF5) and frizzled-related protein (FRZB) were both originally identified from a chondrogenic extract of adult articular cartilage. In joint development, GDF5 is specifically expressed in the joint interzone, where the future joint will form. Mutations in the *GDF5* gene result in severe skeletal malformations, with joint fusions in both mouse and human. In humans, single nucleotide polymorphisms (SNPs) within the *GDF5* gene have been associated with OA susceptibility. In mouse mutants, more severe OA is observed. FRZB function is linked to chondrocyte proliferation during development. SNPs in the human *FRZB* gene have been associated with hip OA. Induced models of OA in mice show increased severity of disease in the absence of the *Frzb* gene.

The goal of OA research is to search for new therapeutic strategies that could prevent, reduce or stop the progression of the disease or, alternatively, resolve the existing damage to the joint. Unfortunately, the development of such interventions is complex and challenging owing to the multifactorial complexity of the disease. Until now, combined efforts from academia and industry have failed to bring disease-modifying anti-OA drugs (DMOADs), with convincing efficacy and reliable safety properties, into daily clinical practice (Bijlsma et al., 2011; Martel-Pelletier et al., 2012). The reason for this failure is partially due to the need for a more comprehensive knowledge of the major pathophysiological factors that contribute to the disease process and progression. However, as described in the following sections, the study of joint physiology and recent advancements in genetic and ‘omics’ technologies have helped identify important players and potential therapeutic targets in OA. As exemplified in Fig. 2, in some cases (which will be described in more detail below), basic knowledge of the processes of joint development and homeostasis has driven subsequent steps in experimental research and revealed key factors associated with OA.

### Learning from joint physiology: emerging targets and drugs

Fundamental knowledge derived from the study of joint development and homeostasis has driven the identification of promising targets for OA treatment. Enzymes, cytokines and growth factors that regulate cartilage differentiation and destruction, subchondral bone remodelling and synovial inflammation are among the most appealing targets, because all those processes play a crucial role in OA.

Biochemical and molecular studies have identified a series of enzymes that play an active role in the breakdown of the extracellular matrix of the articular cartilage during OA. These include matrix metalloproteinases (MMPs) and a disintegrin and metalloproteinase with thrombospondin motifs (ADAMTS) enzymes (Cawston and Young, 2010; Stanton et al., 2005; Troeberg and Nagase, 2012). Although seemingly effective in *in vitro* and *in vivo* preclinical settings, chemical inhibition of such enzymes, which should counteract cartilage loss, has been challenged by safety issues in human clinical trials (Catterall and Cawston, 2003; Clutterbuck et al., 2009).

Growth factors and (stem) cell-based approaches have received a lot of attention for their ability to enhance extracellular matrix synthesis and thereby preserve cartilage or stimulate its repair in OA (Fortier et al., 2011). For example, the bone morphogenetic protein 7 (BMP-7), also known as osteogenic protein 1 (OP-1), is a growth factor that stimulates cartilage and bone formation during embryonic development and postnatal growth (Boon et al., 2011; Chubinskaya et al., 2007), and has shown good promise in preclinical models of arthritis (Badlani et al., 2008; Badlani et al., 2009; Hayashi et al., 2008; Hayashi et al., 2010; Hurtig et al., 2009; Sekiya et al., 2009; Takahashi et al., 2011). Fibroblast growth factor 18 (FGF-18) is another growth factor that plays a central role in skeletal growth and development (Liu et al., 2002; Ohbayashi et al., 2002), and acts as a ligand for the FGF-receptor 3 (FGFR3) pathway (Davidson et al., 2005). In particular, FGF-18 exerts strong anabolic effects on chondrocytes – the unique cell type found in the articular cartilage that synthesizes the characteristic extracellular matrix of the tissue (Ellman et al., 2013; Ellsworth et al., 2002; Moore et al., 2005) – and its capacity to stimulate cartilage repair was demonstrated in a rat meniscal tear model of OA (Moore et al., 2005). In addition to its direct effects on cartilage growth, FGF-18 might also stimulate the BMP signalling by repressing the BMP antagonist Noggin (Reinhold et al., 2004). Currently, both OP-1 and FGF-18 are being tested in OA clinical trials (Hunter et al., 2010; Lohmander et al., 2014). Autologous chondrocyte transplantation has been successfully implemented in the clinical practice for repairing traumatic cartilage lesions (Harris et al., 2010; Minas, 2012), a condition that can predispose to OA; however, this approach is currently not suitable for the treatment of advanced OA-induced cartilage damage because this is too diffuse to approach with current surgical techniques or autologous chondrocyte transplantation (Luyten and Vanlauwe, 2012).

Abnormal remodelling of the subchondral bone is also associated with OA and usually leads to the production of a thicker but mechanically less responsive tissue (Lories and Luyten, 2011). Therefore, targeting subchondral bone metabolism in OA could also be an approach for OA treatment. Until now, strontium ranelate is the only drug reported to have an anti-bone-resorption (or breakdown) effect and at the same time a positive effect on bone formation (Bonnelye et al., 2008; Marie et al., 2011; Tat et al., 2011). Previous studies also suggested an anabolic effect of this drug on cartilage (Henrotin et al., 2001). The beneficial effects of strontium ranelate have been confirmed in OA patients (Alexandersen et al., 2011; Reginster et al., 2013) but, recently, important safety issues related to the use of this agent (i.e. an increased risk of acute coronary syndrome) have been raised (EMEA, 2013). Nevertheless, a recent nationwide cohort study in Denmark did not support an association between strontium ranelate and acute coronary syndrome (Svanström et al., 2014), thus, the safety aspects associated with this drug remain controversial.

Calcitonin, a hormone that regulates calcium homeostasis and bone formation, is another molecule of interest that has been successfully used for the treatment of osteoporosis (Binkley et al., 2012). Osteoporosis is a common skeletal disease characterized by low bone mass and abnormal bone architecture that increase the risk of bone fracture. Drugs in osteoporosis treatment typically target the breakdown of bone by osteoclasts, a process that is out of balance in this disease. Although calcitonin demonstrated a protective effect on joint damage in a rat preclinical model of OA (Nielsen et al., 2011), it failed to prove therapeutic in a Phase III clinical trial (Karsdal et al., 2014). Bisphosphonates, another class of drug that targets bone breakdown by osteoclasts and is widely used for the treatment of osteoporosis, have also been tested in OA but the results remain controversial (Davis et al., 2013; Laslett et al., 2013). Additionally, cathepsin K (a protease responsible for the degradation of bone matrix by osteoclasts) is an interesting drug target because it regulates both cartilage and subchondral bone homeostasis (Connor et al., 2009; Dejica et al., 2012; Dejica et al., 2008; Hayami et al., 2012; McDougall et al., 2010), but its role has not been fully explored in OA. More recently, transforming growth factor-beta [TGFβ; a pleiotropic growth factor that has been extensively studied for its anabolic effects on cartilage (van der Kraan, 2014)] has been considered for OA because of the important effects that this factor elicits on the subchondral bone. Overexpression of TGFβ in osteoblasts (the bone-forming cells) and in the subchondral bone induces OA, whereas inhibition of its signalling pathway prevents OA, suggesting that the inhibition of TGFβ might be a potential treatment strategy for OA (Zhen et al., 2013).

Synovial inflammation is commonly seen in individuals with OA and is characterized by the production and release of proinflammatory cytokines and other inflammatory mediators. At least some of these factors diffuse via the synovial fluid into the superficial layer of the cartilage, where they activate the production of catabolic factors by chondrocytes thereby leading to cartilage destruction. IL-1β and TNFα are two of the major cytokines involved in OA-associated synovial inflammation (Kapoor et al., 2011). In light of this, the inhibition of the IL-1β and TNFα pathways has been tested as a therapeutic strategy in OA. In the case of IL-1β, several approaches have been explored to inhibit its activity, including the block of the IL-1β converting enzyme (ICE), the application of IL-1β receptor antagonists or of soluble receptors, and the use of specific antibodies against this cytokine (Jotanovic et al., 2012). Unfortunately, so far these strategies have failed to demonstrate consistent clinical effects in both hand and knee OA (Chevalier et al., 2009; Cohen et al., 2011; Shin et al., 2013; Yang et al., 2008). Anti-TNFα drugs can successfully treat rheumatoid arthritis. This has triggered interest in assessing these compounds in OA, in particular in hand OA because this form often presents with severe joint inflammation. However, in the past few years, results from independent small clinical trials have questioned the feasibility of this approach because of the lack of clear therapeutic effects for OA patients (Chevalier et al., 2014; Güler-Yüksel et al., 2010; Maksymowych et al., 2012; Verbruggen et al., 2012).

### GFD5 and DOTL1: two promising targets suggested by genetic studies

OA has a complex etiology to which genetic, acquired and environmental factors contribute (Reynard and Loughlin, 2013). The presence of a genetic component in OA is supported by a growing number of sources, including epidemiological and linkage studies, candidate gene approaches, and genome-wide association studies (GWAS). However, all these approaches are complex and still present some difficulties. Indeed, the discovery of genetic susceptibility factors associated with OA has been hindered by the presence of multiple phenotypical manifestations of the disease, which made the definition and classification of patient phenotypes particularly challenging and, thus, limited the power of genetic association analyses (Kerkhof et al., 2011). Candidate gene approaches in OA research have been debated because suggested associations were rarely replicated in multiple population studies (Reynard and Loughlin, 2013). Novel technologies, such as GWAS and meta-analyses on large datasets and multiple cohorts, proved promising for OA and succeeded in identifying regions with multiple candidate genes, for instance a gene cluster on chromosome 7q22 that includes *COG5*, *HBP1*, *DUS4L*, *PRKAR2B* and *GPR22* (Evangelou et al., 2014; Kerkhof et al., 2010; Rodriguez-Fontenla et al., 2014; Styrkarsdottir et al., 2014; Zeggini et al., 2012). However, the role of some of these genes in joint physiology still needs to be fully explored. In this section, we illustrate some of the main challenges beyond genetic association studies, focusing in particular on two potential targets that emerged from these approaches.

Growth and differentiation factor 5 [*GDF5*; also known as cartilage derived morphogenic protein 1 (*CDMP1*)] is a member of the BMP/TGFβ superfamily and was originally identified from a chondrogenic extract of bovine healthy articular cartilage (Chang et al., 1994) (Fig. 2). During joint development, this factor is specifically expressed in the joint interzone [the region in which future joints will form (Storm and Kingsley, 1996)], where it stimulates proliferation and differentiation of chondrocytes. In humans, loss-of-function mutations in *GDF5* cause severe skeletal phenotypes characterized by joint fusions (Polinkovsky et al., 1997; Thomas et al., 1997; Thomas et al., 1996). A 5′ untranslated region (UTR) C/T single-nucleotide polymorphism (SNP) (rs143383) has been associated with susceptibility to OA in the Japanese and Han Chinese population, with this variant resulting in lower levels of *GDF5* expression (Miyamoto et al., 2007). The association between the rs143383 SNP and OA is one of a few that have been confirmed in different ethnic groups and by meta-analysis (Evangelou et al., 2009; Valdes and Spector, 2011). Because of the chondrogenic properties of GDF5, it is not surprising that genetic variations leading to decreased levels of its expression can affect cartilage growth and contribute to OA. However, the role of GDF5 seems much more complex and alterations of its function might also affect other tissues of the joint. For example, GDF5 deficiency in mice results in abnormal ligament laxity and could thereby contribute to OA development by reducing joint stability (Daans et al., 2011). In addition, GDF5 might also affect subchondral bone remodelling, as suggested by the fact that reduced GDF5 levels are associated with an abnormal structure of the collagen fibres of the bone (Daans et al., 2011). GDF5 has not been used as a pharmaceutical target up until now, but in principle it could be considered as a factor that stimulates cartilage extracellular matrix synthesis and could strengthen ligaments.

As highlighted above, GWAS studies in OA have been hampered by the heterogeneity of disease phenotypes, which has also guided the search for alternative measures of disease outcomes (disease ‘endpoints’) to be used for the identification of potential genetic associations. An example of such an approach is represented by an OA GWAS in which the hip joint space width was used as a measure of cartilage thickness (Castaño Betancourt et al., 2012). This study identified a genetic variant in the *DOT1*-like histone H3 methyltransferase (*DOT1L*) gene that was robustly associated with increased joint space width and reduced risk for hip OA in Caucasian populations. DOT1L is an evolutionarily conserved enzyme and the only known H3K79 methyltransferase (Nguyen and Zhang, 2011). Originally identified in yeast telomere silencing (Singer et al., 1998), it functions as a positive mediator of gene transcription and DNA repair, and as a regulator of the cell cycle (Nguyen and Zhang, 2011). In cancer, oncogenic fusion proteins caused by chromosomal rearrangements have been linked to DOT1L-mediated leukemogenesis (Mueller et al., 2007). The *DOT1L* association is attractive for directing future therapeutic strategies in OA based on different considerations. First, DOT1L is an enzyme and, thus, can be targeted by pharmacological agents (Barry et al., 2010), with DOT1L inhibitors already being tested in cancer clinical trials (Daigle et al., 2011; Deshpande et al., 2012). Moreover, DOT1L was shown to interact with the Wnt signalling cascade (Castaño Betancourt et al., 2012; Mahmoudi et al., 2010), a molecular signalling pathway that plays essential roles in joint and bone development and which has been extensively studied in the last decade and proposed as a potential target in OA (Lories et al., 2013). Unfortunately, direct targeting of Wnt signalling proteins has been difficult because recombinant Wnts are difficult to obtain mainly due to solubility issues; additionally, Wnt signalling has also been associated with cancer, thereby raising concerns about intervening within this pathway. These obstacles could be overcome by using DOT1L as an alternative target. Finally, genetic variants in both the DOT1L (Sovio et al., 2009) and other OA susceptibility genes (Sanna et al., 2008) have also been associated with variations in human height, further supporting the involvement of factors that regulate bone growth in the disease.

### Target discovery based on ‘omics’ technologies

Transcriptomics, proteomics and epigenomics respectively study and map the RNAs, proteins and epigenomic modifications of different cell types and tissues from human and model organisms. These datasets can be compared between healthy and disease states to better understand the mechanisms underlying a disease and to guide the development of new therapeutic strategies, a principle that also applies to OA. Transcriptomics has already been shown to be a powerful tool to gain knowledge of the onset and progression of OA. For instance, transcriptome profiles of cartilage from wild-type mice and mice lacking ADAMTS5 (an aggrecan-destructive enzyme associated with OA, as already mentioned above) with surgically induced OA identified several genes (e.g. *Ptgs2*, *Crlf1*, *Padi2* and *Col8a2*) that might contribute to OA initiation and whose role seems independent of ADAMTS5 activity (Bateman et al., 2013). Previously, our group performed a microarray of transcripts obtained from the articular cartilage and subchondral bone of wild-type and Frzb-knockout (*Frzb^−/−^*) mice (Lodewyckx et al., 2012). Frizzled-related protein (FRZB) is a secreted antagonist of the Wnt signalling pathway (Wang et al., 1997) and was originally discovered in a chondrogenic extract of articular cartilage and linked to chondrocyte proliferation (Hoang et al., 1996) (Fig. 2). Polymorphisms in the human *FRZB* gene were associated with hip OA in a Caucasian population (Loughlin et al., 2004). Our observation that other Wnt antagonists show compensatory upregulation in the absence of FRZB in the knockout model supports the concept that the Wnt signalling pathway is tightly regulated in the joint while also emphasizing the molecular complexity of the Wnt signalling pathway in the regulation of joint homeostasis (Lodewyckx et al., 2012).

Gene or RNA expression levels cannot predict the exact amount and the level of activity of proteins that are produced in a cell at a given time, because these are both strongly influenced by processes of alternative splicing, post-translational modifications and protein degradation. Therefore, proteomics offers the possibility to describe what might be truly happening in a tissue in terms of its protein repertoire and content. Proteomic analyses performed on both *in vitro* and *ex vivo* systems, including cultured chondrocytes, osteoblasts, synoviocytes or cartilage explants, have already contributed to the identification of potential drug targets. For example, combined transcriptomic and proteomic analyses of synovial fluids and membranes from OA patients showed elevated levels of activation of the complement pathway, a series of proteins that have effector functions in the innate immune system (Wang et al., 2011). These data were confirmed by *in vivo* experiments with mice deficient in complement component 5 (C5), C6 or the complement regulatory protein CD59a (Wang et al., 2011). Absence of complement components protected against OA development in different induced mouse models of OA. In particular, absence of C5 reduced the amount and effects of inflammatory and tissue-destructive molecules in the joint. Blocking of complement activation in the joint could therefore be considered a therapeutic target and merits further attention.

Additionally, large-scale screening of proteomic datasets can be used to search for potential OA-related biomarkers in body fluids that are rich in protein content, such as blood plasma and serum, urine and synovial fluid. The identification of reliable biomarkers for OA would help optimize the diagnosis of the disease and monitoring its progression.

Epigenetic changes are defined as heritable stable alterations in the expression of a gene that occurs without modifications of its DNA sequence. DNA methylation, histone modifications and RNA-associated gene silencing [induced by small non-coding microRNAs (miRNAs)] are the three basic mechanisms that play crucial roles in epigenetic gene regulation, in both physiological and pathological conditions. Epigenetic alterations can also influence the expression of OA susceptibility genes. For example, DNA methylation was shown to regulate the allelic expression imbalance of rs143383 (Reynard et al., 2011), an SNP of the *GDF5* gene that is associated with a higher risk of OA, as mentioned above. Epigenetic alterations might also represent a feasible target for drug therapy. Indeed, intra-articular injections of trichostatin A, a small molecule that inhibits histone deacetylases (HDACs), decreased cartilage damage in a surgically induced OA model in rabbits (Chen et al., 2010). In addition, miRNAs seem to play an important role in OA, as suggested by a study showing that the expression of miRNA-140, which increases during normal chondrocytic differentiation of mesenchymal stem cells, is reduced in human articular cartilage of OA patients, likely in response to IL-1β (Miyaki et al., 2010), one of the OA-associated cytokines. Until now, epigenetic approaches in OA have focused primarily on the analysis of single genes. However, with ‘omics’ technologies in continuous expansion, a whole-genome epigenetic fingerprint analysis of OA tissues might become feasible in the near future.

## *In vitro* models of the joint

*In vitro* models are important tools to elucidate the molecular mechanisms and pathways that are involved in joint physiology and pathophysiology. These models include *ex vivo* tissue samples, primary cell cultures and cell lines but also more complex setups that allow the study of the interaction between different cells and tissues within the joint. In this section, we will review pros and cons of *in vitro* systems of the joint with a particular focus on cartilage models.

### *In vitro* models of the cartilage

The articular cartilage has a key structural and functional role in joint physiology and disease; therefore, a high number of *in vitro* models have been developed to study this specialized tissue in normal and pathological conditions. In *ex vivo* tissue systems the cartilage is isolated from the rest of the organism and can be tested in experimentally controlled conditions to detect specific pathological hallmarks via biochemical and immunohistochemical techniques. Potential disadvantages of this approach include the descriptive nature of the data collected limiting the mechanistic insights that can be obtained, the fact that the tissue of interest is isolated from potential systemic influences, and difficulties associated with defining positive and negative controls, in particular the ethical considerations when normal cartilage is required. More functional studies can be performed using *ex vivo* tissue cultures. For example, slices of human osteoarthritic cartilage or mice femoral head caps can be kept in culture to allow the analysis of their protein secretion profile in the presence or absence of specific factors that can mimic an OA-associated environment (Liu-Bryan and Terkeltaub, 2010; Ozaki et al., 2004). These models have several drawbacks, such as a limited availability of tissue and high inter-experimental variability. However, they have been useful to elucidate the contribution of different tissue-destructive enzymes in cartilage degradation, e.g. MMP-3 and MMP-13 (Liu-Bryan and Terkeltaub, 2010). Tissue explants, but also cells cultured in hydrogels, can be used to mimic the behaviour of the cells in an extracellular-matrix-like environment in biomechanical loading experiments, thereby assessing the effects of mechanical strain on chondrocytes (Bryant et al., 2004; Quinn et al., 1998; Treppo et al., 2000).

Primary articular chondrocytes isolated from animal or human donor cartilage tissue can also be used as an *in vitro* model. The main issue with this type of culture is that chondrocytes rapidly lose their molecular signature when taken out of the joint environment and quickly dedifferentiate (Abbott and Holtzer, 1966; Holtzer et al., 1960). High-density pellet cultures or embedding of the cells in agarose can be an option to maintain the chondrocyte phenotype in culture at least to some extent (Häuselmann et al., 1994; Tew et al., 2008). However, as discussed in more detail below, this feature of primary cultures could also be an advantage, because dedifferentiation and phenotypical changes are often associated with OA. Clear disadvantages of primary cells include the difficulty to obtain a sufficient number of cells, the diversity of cells within the cultures as compared to other cell lines, and the fact that the cells are not easy to transfect, thereby limiting the possibility to alter the expression of target genes (via loss- or gain-of-function mutations) to elucidate their role in OA. In addition, appropriate and reliable control tissues are limited because healthy human articular chondrocytes are hard to obtain because of ethical considerations, whereas chondrocytes isolated from non-pathological subregions of OA joints might already show signs of disease at the molecular level, such as reduced collagen type II and aggrecan expression (Squires et al., 2003). Indeed, articular chondrocytes have a very specific molecular profile (for instance, they typically express type II collagen) that clearly differs from the profile of chondrocytes found in other postnatal cartilaginous tissues such as the epiphyseal, nose and ear cartilages (Dell’Accio et al., 2001). However, under pathological conditions, articular chondrocytes seem to lose their phenotypical stability, they start to dedifferentiate or differentiate towards hypertrophy, and can eventually undergo necrosis or apoptosis (Lotz and Loeser, 2012). Cartilage hypertrophy is characterized by a switch in the production of the extracellular matrix proteins (from collagen type II to type X) and can be accompanied by phenomena of matrix mineralization (Lefebvre and Bhattaram, 2010; Lotz and Loeser, 2012). These processes normally occur during cartilage development and growth but can be deleterious for the articular cartilage in the adult. The molecular mechanisms regulating the phenotypical changes associated with OA are believed to recapitulate at least some of the differentiation steps that take place during cartilage development. Therefore, signalling pathways that are typically activated during joint development, such as TGFβ, BMP, WNT and FGF have been investigated in chondrocyte culture models of OA (Lories, 2011). Interestingly, as reported previously in the text, a number of potential OA susceptibility genes have emerged from the study of these pathways (as extensively reviewed in Reynard and Loughlin, 2013).

The mechanisms and the molecular signatures that characterize chondrocyte differentiation and hypertrophy can be also modelled in immortalized clonal cell lines. In these lines, cells can be produced in large amounts and grown indefinitely, offering an ideal tool to explore molecular and biochemical processes. For example, the ATDC5 cell line, derived from mouse teratocarcinoma cells, is a chondrogenic cell line that undergoes sequential stages of differentiation analogous to the ones that are recognized during cartilage development. Therefore, this cell line represents a well-established model to study the factors and signalling pathways that influence cell behaviour during the processes of chondrogenesis, chondrocyte differentiation and terminal differentiation to hypertrophy (Atsumi et al., 1990; Newton et al., 2012). Gain- or loss-of-function mutations of specific genes can also be easily induced by cell transfection: stable cell lines can be established within a month of transfection. In addition, ATDC5 cells can be cultured in monolayer, in three-dimensional (3D) micromasses/pellets or in agarose beads. The use of 3D cultures is particularly advantageous in experimental setups because a 3D matrix structure of collagens and proteoglycans favours the expression of the normal phenotype of the cartilage and the 3D environment also allows the formation of cell-matrix interactions, which are essential during chondrogenesis (Castaño Betancourt et al., 2012). These important features are obviously not reproducible in monolayer cell cultures. Recent studies on the role of hypoxia induced factor-2a (HIF2a) and DOT1L (Castaño Betancourt et al., 2012; Saito et al., 2010) illustrate the potential of such a standardized system: HIF2a was demonstrated to stimulate chondrocyte hypertrophy and DOT1L shown to be essential in early cartilage differentiation.

Other cell lines of interest include human articular cartilage immortalized cell lines (Goldring et al., 1994), among which the C-28/I2 line seems particularly valuable for the study of cartilage physiology. In fact, this cell line expresses a high level of matrix-associated molecules that are involved in both cartilage anabolic and catabolic processes (Finger et al., 2003). Detailed analysis demonstrated that human cartilage immortalized cell lines show essential cartilage-specific features, but further investigations are necessary to evaluate whether they can reliably substitute primary chondrocyte cells (Finger et al., 2004; Finger et al., 2003). This limitation also applies to other chondrocyte cells lines, including ATDC5 cells.

Finally, stem cell lines are particularly attractive because of their potential therapeutic use in regenerative medicine and have been explored also for applications in OA. Controlled induction of induced pluripotent stem cells (iPSCs) has been used as a tool to reprogramme dermal cells in order to obtain large amounts of cartilage-like tissue, which could be used for cartilage repair (Hiramatsu et al., 2011). Mesenchymal stem cells are multipotent stem cells that give rise to multiple connective tissue lineages, including cartilage and bone. These cells have been isolated from different human or animal tissues such as bone marrow, adipose tissue and synovium. *In vitro* analyses and *in vivo* cell differentiation assays using xeno-cell transfer in immunodeficient mice showed that optimization of culture media and stimulation with growth factors is necessary to obtain the desired cell differentiation in these cells (De Bari et al., 2001; Eyckmans et al., 2012; Pittenger et al., 1999). Current limitations to the use of stem cells for OA are numerous and include challenges in defining optimal cell populations in patient selection and in surgical methods required for proper cell transfer (for an extensive discussion see Luyten and Vanlauwe, 2012).

### Other *in vitro* systems: the bone and the synovial tissues

Bone remodelling is an active and dynamic process that is finely regulated by catabolic and anabolic reactions, through which bone components are constantly broken down and built up. Dysregulation of bone homeostasis can lead to several skeletal pathologies, including OA, which is often associated with alterations in the subchondral bone turnover (Karsdal et al., 2014). As described above for the cartilage, several *in vitro* models can be applied to study bone remodelling in OA and these include *ex vivo* tissues, cell cultures and lines, and also more complex tissue culture systems that allow the investigation of the interactions between bone and cartilage cells. Several pre-osteoblastic and osteoblastic cell lines are available and can be used to study the differentiation of bone-forming cells (Czekanska et al., 2012). The process of osteogenesis can also be investigated in *in vitro* osteogenic assays using bone-derived cells originating from three different primary cell sources: (1) fetal or neonatal calvarial cells (cells that form the upper portion of the skull), (2) mesenchymal stromal cells, including bone-marrow-derived multipotent mesenchymal stromal cells (BMSCs) and periosteum-derived cells (PDCs) or (3) cells migrating from trabecular bone explants (Czekanska et al., 2012). Some evidence suggests that cells derived from OA bone have a different molecular profile as compared to healthy controls. For instance, in a series of experiments, Sanchez et al. demonstrated that the gene-expression profile of OA sclerotic subchondral bone osteoblasts was characterized by increased levels of MMP13, COL1A1 and COL1A2, osteopontin, alkaline phosphatase, osteocalcin, and vascular endothelial growth factor (Sanchez et al., 2008).

The cross-talk between chondrocytes and subchondral bone cells seems essential to optimize the structure of the interface between the hard mechanoresistant bone and the softer load-dissipating articular cartilage. Molecular and cellular interactions between chondrocytes and osteoblasts can be analyzed in a number of *in vitro* systems. For example, Sanchez and co-authors used a co-culture model in which chondrocytes were grown in inserts with a micro-pored membrane upon a monolayer of osteoblasts isolated from OA or control subchondral bone (Sanchez et al., 2005). In this system, the presence of an insert allows the exchange of factors and molecules between the cell layers and, afterwards, individual analysis of chondrocytes, osteoblasts and of the conditioned medium can be performed. In these co-cultures, osteoblasts isolated from sclerotic zones of subchondral OA, but not those isolated from non-sclerotic regions, could induce phenotypic changes in OA chondrocytes leading to initiation of hypertrophic differentiation and subsequent matrix mineralization (Sanchez et al., 2005). Therefore, these experiments strongly support the key role of bone-cartilage cross-talk in driving OA pathological processes.

Synovitis is increasingly recognized as an important contributor to the development and progression of OA. Therefore, synovial tissue explants, synovial cell cultures and synovium-cartilage co-cultures have been employed as models to elucidate the role of the synovium in OA and to identify potential mechanisms relevant to the disease (Lee et al., 2013; van Buul et al., 2012). For instance, Lee et al. demonstrated that co-cultures of normal synovium and injured cartilage are associated with a protective effect of synoviocytes by reducing the incidence of both focal cell loss and chondrocyte cluster formation, two major hallmarks of OA.

## *In vivo* models of OA

*In vivo* models aim to recapitulate OA-associated processes and lesions in the whole animal. Reproducing features of OA in animal systems is crucial to gain a better understanding of disease mechanisms and to assess response to potential therapies, which is a prerequisite for translating basic findings into therapies for patients. Animal models of OA include naturally occurring OA in (experimentally accelerated) aging, transgenic models, and surgically or chemically induced OA (summarized in Tables 1 and 2). Unfortunately, none of these models can fully reproduce the features and symptoms of human OA, owing to the complexity and heterogeneity of the disease. Each model and species has its own advantages and disadvantages (summarized in Table 1), and the impact of preclinical research depends largely on the choice of the most appropriate model of OA for the specific process that is under investigation. For example, spontaneously occurring OA models (which include aged animals or transgenic animals that develop OA as an effect of a transgene) allow following the development of OA from the early to the late stages, but are relatively costly and time consuming and present with more variability in the disease phenotype. By contrast, in surgical or chemical models of OA, the disease can be rapidly induced and its manifestations are less variable, but a limitation of these models is that they more reflect post-traumatic OA alterations rather than spontaneous changes occurring in human OA. Small animals (mice, rats, rabbits and guinea pigs) (Table 1) are most often used to investigate specific disease mechanisms and for initial drug screenings for reasons of cost-effectiveness, ease of handling and housing, and opportunity for genetic manipulations. Large animal models (Table 1) show more similarity to humans in terms of cartilage morphology, joint anatomy and joint biomechanical function, and thus provide more clinically relevant data. However, these models are relatively expensive, present important ethical concerns and offer limited possibility of genetic manipulations. Nevertheless, they are a crucial preclinical system to validate potential therapeutic strategies. Most animal models of OA are focused on the joint of the knee as this is the main affected joint in human OA and its size is sufficiently large to allow intra-articular manipulations even in small animals.

**Table 1.**
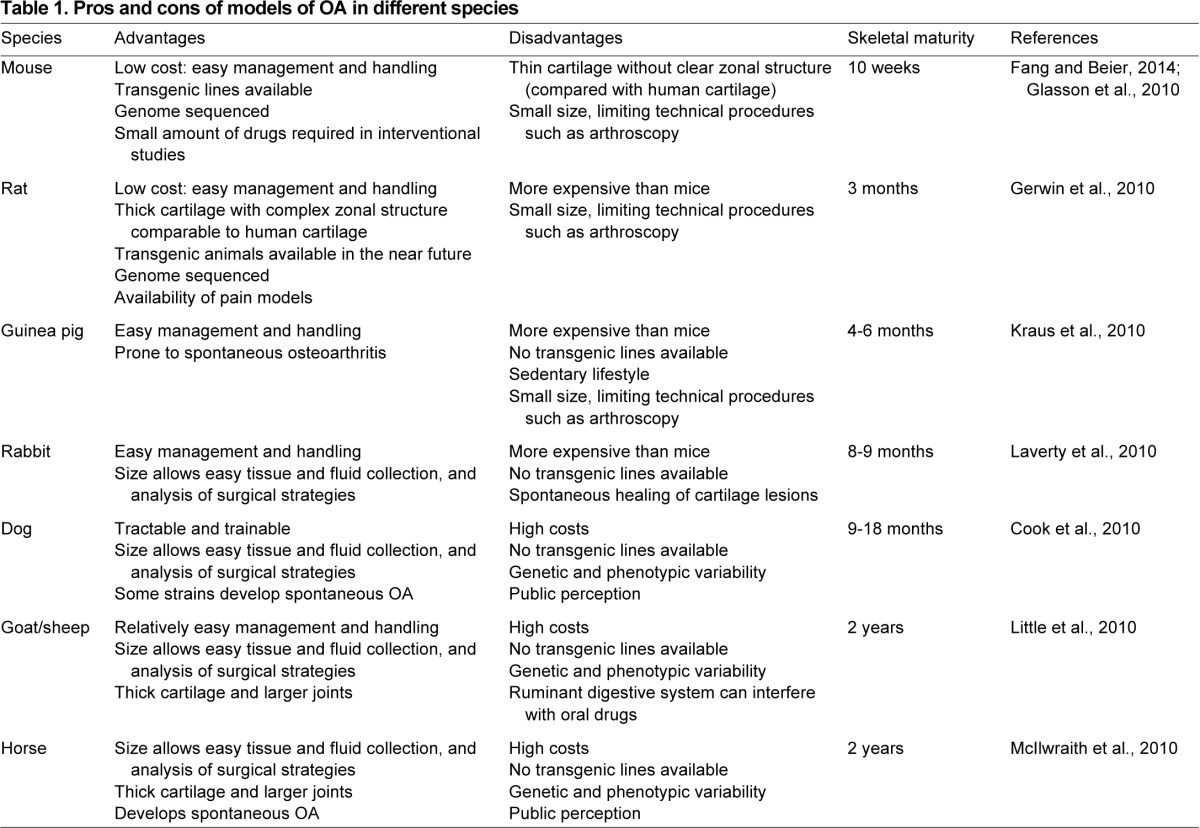
Pros and cons of models of OA in different species

**Table 2.**
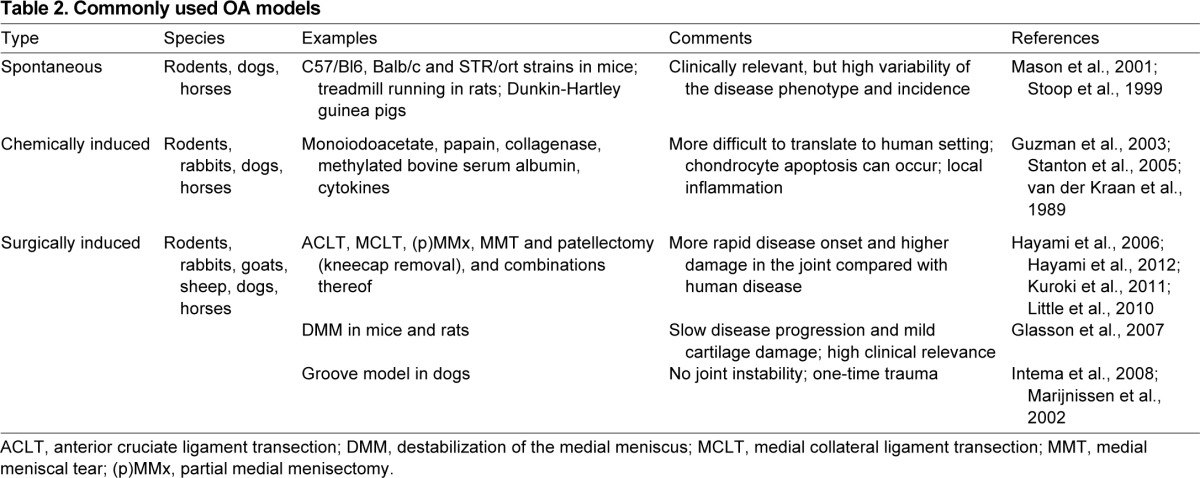
Commonly used OA models Small animal models

### Small animal models

Mice are widely used as a research model because of the easy management, low costs and the availability of genetically modified lines. Originally, C57Bl/6 and BALB/c mice were widely employed for the study of OA because aged animals of these strains develop OA spontaneously (Stoop et al., 1999). However, more recently the STR/ort mouse strain was discovered, which showed a more pronounced form of OA (Mason et al., 2001); therefore, this strain is now more often used in OA research, especially for drug screening studies. The possibility to induce a wide range of genetic modifications (for example, via the transgenic expression of a gene, knock-out and knock-in strategies, inducible and tissue-specific gene expression) makes mouse models an extremely useful tool for replicating OA-associated genetic defects in the animal and elucidating the molecular players that are involved in the pathogenesis of the disease. For example, the crucial role of catabolic enzymes such as MMP13 in OA was suggested by a study in which the development of spontaneous articular cartilage damage occurred after constitutive cartilage-specific overexpression of MMP13 in mice (Neuhold et al., 2001). In some cases, mouse genetics revealed complex interactions existing between a given factor and OA. For instance, both cartilage-specific overexpression of β-catenin (a crucial mediator of Wnt signalling) and of its endogenous inhibitor led to OA development in mice, demonstrating that a single pathway can contribute to OA through different mechanisms (Zhu et al., 2008; Zhu et al., 2009).

**Fig. 3.**
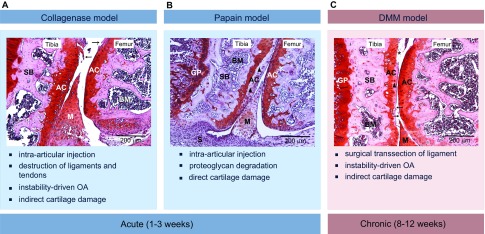
**Examples of *in vivo* mouse models of induced OA of the knee, with typical histological appearance and main features.** Each panel shows a representative frontal hematoxylin–safranin-O-stained section of a mouse knee joint (medial condyle) with clear histopathological alterations: arrows indicate cartilage fibrillation, triangles indicate loss of proteoglycans and asterisks indicate loss of cartilage. (A) Intra-articular injections of collagenase are used to induce acute (within 1–3 weeks) damage to the ligaments and tendons of the knee. This will subsequently lead to secondary cartilage damage. (B) Intra-articular injection of papain leads to acute direct cartilage damage, mainly characterized by an obvious loss of proteoglycans. (C) Destabilization of the medial meniscus (DMM) is a more clinically relevant model in which dissection of the medial meniscus leads to destabilisation of the joint. After this surgical induction, mice develop mild to severe histological features of OA within 8 weeks. Scale bars: 200 μm; original magnifications 10×. AC, articular cartilage; BM, bone marrow; GP, growth plate; M, meniscus; S, synovium; SB, subchondral bone.

Chemically and surgically induced models of OA in mice can be obtained via different approaches. Intra-articular injections of enzymes such as collagenase (van der Kraan et al., 1989) and papain (van der Kraan et al., 1989) are commonly used to induce acute OA, and are characterized by injury to the knee ligaments (leading to severe joint instability and rapid cartilage breakdown) or by direct enzymatic damage to the cartilage extracellular matrix (also triggering further rapid cartilage breakdown), respectively (Fig. 3). Inflammation-associated OA can be modelled by injections of methylated bovine serum albumin (mBSA) (Stanton et al., 2005), which triggers an acute inflammatory reaction in the joint. In this model, different cytokines and infiltrating inflammatory cells have a catabolic effect on the articular cartilage. Destructive surgical models include partial medial meniscectomy (PMM), medial collateral ligament transection (MCLT) and anterior cruciate ligament transection (ACLT) (Clements et al., 2003; Hayami et al., 2012). These models lead to severe OA pathology in mice and were developed as translational models because meniscus injuries and cruciate ligament injuries predispose to OA in humans. Finally, the destabilization of the medial meniscus (DMM) surgical model (Glasson et al., 2007) is a frequently used chronic model of OA in mice, with a high clinical relevance (Fig. 3). The transection of the medial menisco-tibial ligament alters the mechanical stability of the knee joint and induces cartilage damage; these alterations closely resemble some of the changes that occur in progressive human OA (Glasson et al., 2007). Mouse models are more extensively reviewed elsewhere (Fang and Beier, 2014).

Mice are not an ideal system to model the alterations of the biomechanical function of the joint that are associated with human OA due to their small size as compared to humans. In addition, in mice the cartilage of the knee has relatively few cell layers and a reduced zonal tissue organization as compared to larger species, which makes it difficult to mimic small lesions that slowly and progressively extend through the different non-calcified layers of cartilage, as reported in human OA (Glasson et al., 2010). Therefore, these aspects can be better characterized in larger rodent and non-rodent animal models.

Like mice, rats are relatively low-cost laboratory animals that are easy to house and handle, but, unlike mice, naturally occurring OA is extremely uncommon in rats and, although the technology to generate transgenic and knockout lines is advancing in other fields (Huang et al., 2011), transgenic rat models of OA are not yet available. Nevertheless, rats possess a thicker cartilage with a complex zonal structure, which makes partial and full cartilage lesions possible to reproduce (Gerwin et al., 2010) via both chemical [injection of iodoacetate (Guzman et al., 2003)] or surgical [medial meniscal tear (MMT) or ACLT transections (Hayami et al., 2006)] approaches. Rats are therefore an attractive animal model to test cartilage repair strategies such as gene therapy, stem cell transplantation, chondrocyte implantation and local growth factor stimulation. Rat models have also been used for the assessment of OA-related pain (Bove et al., 2006) and of novel OA pain therapies (Ahmed et al., 2012; Cowart et al., 2012; Schuelert and McDougall, 2006).

Other small animal models of OA include rabbits (Laverty et al., 2010) and guinea pigs (Kraus et al., 2010). The knee joint of the rabbit shares some anatomical similarities with the human knee but, because its biomechanical function is very different, rabbit models are less suitable for functional studies. However, the larger size of the joints as compared to mice has triggered interest in this species as a source of cells for *in vitro* expansion and cell transfer studies. Therefore, rabbit models of cartilage lesions are often used to test cartilage repair strategies (Reyes et al., 2013). However, a disadvantage is that young rabbits have a high potential for spontaneous healing of cartilage and osteochondral lesions, thereby limiting the translational value of the model. Finally, Hartley guinea pigs are a frequently used model of OA because they develop OA spontaneously and display progressive degenerative changes that closely resemble the development of the disease in humans. The guinea pig model has clear time-effectiveness advantages as compared to large animal systems because the time of skeletal maturation is much shorter in this species (Kraus et al., 2010). Bone growth ceases by 4 months, although closure of the growth plates in femur and tibia is only seen after 7 and 12 months, respectively. However, early spontaneous changes in cartilage and bone, much alike human OA, can be seen as early as 2 months after birth and increase gradually until 18 months (Kraus et al., 2010).

### Large animal models

A major advantage of using large animals is that they share more similarities with humans in terms of macroscopic and microscopic anatomy and, owing to their large size, topographical analysis of joint cartilage by arthroscopy and serial aspiration of synovial fluid for biomarker analysis are possible. In addition, these models are also very useful for *ex vivo/in vitro* investigations because large amounts of tissue samples and cells can be obtained. A disadvantage is that histological analyses of large animal joints are more complex because the whole joint cannot be captured on a single microscope section.

The canine model is a valuable model for biomarker studies in OA. Dogs are prone to develop naturally occurring OA with overuse or age and receive similar treatments as humans (Cook et al., 2010; Tirgari and Vaughan, 1975). Synovial fluid from OA-affected dogs contains various MMPs, degradation products and cytokines that are found in human samples, and these factors have been correlated to cartilage breakdown and inflammation (Pelletier et al., 2005; Pelletier et al., 2004). The most frequently used OA models in dogs are the surgical ACLT (also known as Pond/Nuki model) (Kuroki et al., 2011) and the groove (Intema et al., 2008) models. These models show classical signs of OA, in particular progressive damage to the articular cartilage, but the groove model is induced without joint instability and is therefore expected to be more sensitive to treatment because progression of the disease is milder and dependent on fewer variables (Marijnissen et al., 2002).

Goats and sheep do not develop spontaneous OA (Little et al., 2010), but surgically induced models are available and include partial or total meniscectomy and the ACLT model, which, however, only induces very mild cartilage damage (Little et al., 2010). These large animals are particularly useful for the evaluation of surgical interventions. In addition, the anatomy and in particular the thickness of their cartilage more closely resemble those of human cartilage as compared to rodents. A disadvantage of these models is that ruminants have a peculiar digestive system that can alter the bioavailability of drugs and, thus, they are not very suitable for testing orally administered therapies.

## Measures of disease outcome in animal research

The assessment of joint damage, in particular of cartilage loss, is an essential aspect in animal research and preclinical drug testing. Pioneering work to define criteria for the histopathological assessment of OA was performed by Collins (Collins, 1939), who established a grading system (Grades I–IV) based on the degree of fibrillation of articular cartilage, defined as fraying of its smooth surface. In 1971, Mankin (Mankin, 1971) developed a microscopic histologic histochemical grading system (HHGS; 14-point scoring system) based on the evaluation of a range of cellular changes, extracellular matrix alterations as revealed by staining with Safranin O, and structural changes. Although these scoring systems are still in use, they have many limitations, particularly concerning their potential for the assessment of early OA. This guided the efforts from both academic and industrial OA researchers for developing a standard histological grading system that could advance data interpretation across distinct model systems and provide better criteria for evaluating the outcome of OA treatments. Therefore, the Osteoarthritis Research Society International (OARSI) histopathology initiative (Berenbaum, 2010) defined a standardized scoring system for the most important small and large animal models in which joint degeneration is assessed based on specific evaluation of both depth and extent (surface) of lesions. Ideally, macroscopic and microscopic evaluation of OA features should be extended to non-cartilaginous structures given their important contribution to the disease process. In this regard, the OARSI histopathology initiative filled the gaps of previous scoring systems and provided additional scoring parameters to assess alterations in the synovium, subchondral bone, menisci, tendons and ligaments (Cook et al., 2010; Gerwin et al., 2010; Glasson et al., 2010; Kraus et al., 2010; Laverty et al., 2010; Little et al., 2010; McIlwraith et al., 2010). As compared to cartilage scoring systems, analysis of the other tissues of the joint might still suffer from lack of standardized, validated and comprehensive methodologies. Because these methodologies are still semi-quantitative and partially affected by inter- and intra-observer variability, they are often supported by histomorphometric analyses, which can more reliably quantify cartilage thickness, proteoglycan content, subchondral bone size and synovium width (Pastoureau et al., 2010).

As an alternative to histopathological analyses, which are a time-consuming method, non-invasive imaging techniques such as magnetic resonance imaging (MRI) and micro computed tomography (microCT) are becoming popular in OA animal studies. Non-invasive *in vivo* imaging can be used for longitudinal followup in the same animal to monitor the disease progression and the response to treatments in drug trials. Standard clinical MRI (1.5 and 3T) is being used in large animal models (dogs, goats, sheep and horses) to assess OA-associated alterations in subchondral bone marrow, synovial fluid volume and soft tissue structures such as the articular cartilage and the synovium (Galindo-Zamora et al., 2013; Ley et al., 2013). Rodents and rabbits are too small to undergo standard *in vivo* MRI but, recently, high-resolution micro-MRI (>7T) has been developed for these species to monitor cartilage lesions in surgically induced models of OA (Batiste et al., 2004; Goebel et al., 2010). This technique needs further optimization and is still challenging because it requires the use of specific magnetic coils. Micro-CT is also available but can be used only for high-resolution imaging of bone architecture alterations. Indeed, owing to its low soft-tissue contrast, direct imaging of the cartilage is not feasible with this approach. However, the combination of contrast agents, such as ioxaglate and hexabrix, which allow the visualization of the cartilage, with nano-CT might represent an alternative to conventional microscopy (Kerckhofs et al., 2013).

Biomarker analysis is another non-invasive approach to assess cartilage or bone turnover, as well as synovitis in OA. Serum and urine samples contain biomarkers such as aggrecan and collagen neoepitopes that can be considered as breakdown products of the cartilage extracellular matrix and that could help predict the efficacy of new drugs in animal models (Kraus et al., 2011; Rousseau et al., 2010). Unfortunately, these biomarkers are highly diluted in the blood plasma and in the urine, and exhibit large inter-individual variations, thus requiring highly sensitive techniques.

Pain-related outcomes are beyond the scope of this article but mechanisms of pain in OA, including methods for assessing pain in animal models, have been recently reviewed elsewhere (Malfait et al., 2013).

## Concluding remarks and future directions

OA research offers a range of valid approaches that are currently contributing to advancing our understanding of the disease. The different *in vivo* and *in vitro* models of disease discussed in this Review have contributed in different ways to bring insights into the disease process. Some of these discoveries, including the essential role of growth factors, the identification of key tissue-destructive enzymes and, more lately, the recognition that inflammation is also an important contributor to OA, have suggested potential novel therapeutic targets for OA and support the need to develop new and effective strategies. However, in a translational view, taking basic knowledge from bench to bedside is still a big challenge. One main problem is related to the lack of a ‘holistic’ view of the disease: the focus of OA research should be shifted from an approach solely considering the articular cartilage to a system biology approach, based on the study of the different components of the joint and of their interactions, in order to better understand mechanisms of whole joint failure in OA. In addition, a better definition of disease stages is highly needed. It is becoming clear that early and established OA likely represent heterogeneous diseases, and efforts are ongoing to define classification criteria to differentiate these stages. This would guide more appropriate and timely therapeutic interventions, which could allow more efficient interference with disease progression, as seen in other joint diseases such as rheumatoid arthritis (Luyten et al., 2012).

Finally, one of the biggest challenges in OA is related to heterogeneity of the clinical presentations of the disease. Multiple subsets of patients can exist, with very different molecular, clinical and epidemiological profiles, which makes phenotyping of OA patients particularly challenging. In cohort studies and clinical trials, OA is still commonly defined and classified based on conventional radiographs. Such an approach does not consider and integrate emerging important information about disease mechanisms that is becoming available from the application of novel ‘omics’ technologies and advanced bioinformatic approaches. Other important features that should be taken into account in the classification of patient subsets include the type of OA-associated pain (for example, pain directly attributable to the joint tissue, pain associated with the phenomena of sensitization such as neuropathic pain, psychologically mediated pain), the type of bone reaction [OA hypertrophic versus atrophic bone reaction (Castaño Betancourt et al., 2013)], the identification of genetic susceptibility factors, and the presence or absence of a main causative mechanism (metabolic-driven OA, inflammation-driven OA, mechanically driven OA). Redefining the number of parameters and classification criteria that are used for patient phenotyping would likely help identify patients at a high or low risk to develop severe OA, predict responders and non-responders to a specific intervention, and would hopefully guide the development of personalized treatments, with a high rewarding impact on both patient life and society.
